# Artificial intelligence for optimizing benefits and minimizing risks of pharmacological therapies: challenges and opportunities

**DOI:** 10.3389/fdsfr.2024.1356405

**Published:** 2024-03-18

**Authors:** Salvatore Crisafulli, Francesco Ciccimarra, Chiara Bellitto, Massimo Carollo, Elena Carrara, Lisa Stagi, Roberto Triola, Annalisa Capuano, Cristiano Chiamulera, Ugo Moretti, Eugenio Santoro, Alberto Eugenio Tozzi, Giuseppe Recchia, Gianluca Trifirò

**Affiliations:** ^1^ Department of Medicine, University of Verona, Verona, Italy; ^2^ Department of Diagnostics and Public Health, Verona, Italy; ^3^ Roche Spa, Monza, Italy; ^4^ Digital Transformation Area, Farmindustria, Roma, Italy; ^5^ Department of Experimental Medicine, University of Campania “Luigi Vanvitelli”, Naples, Italy; ^6^ Unit of Research in Digital Health and Digital Therapeutics, Department of Clinical Oncology, Istituto di Ricerche Farmacologiche Mario Negri, IRCCS, Milan, Italy; ^7^ Predictive and Preventive Medicine Research Unit, Bambino Gesù Children’s Hospital, Istituto di Ricovero e Cura a Carattere Scientifico, Rome, Italy; ^8^ daVi DigitalMedicine Srl, Verona, Italy; ^9^ Fondazione Tendenze Salute e Sanità, Verona, Italy

**Keywords:** artificial intelligence, machine learning, pharmacological therapies, pharmacovigilance, pharmacoepidemiology, real-world evidence

## Abstract

In recent years, there has been an exponential increase in the generation and accessibility of electronic healthcare data, often referred to as “real-world data”. The landscape of data sources has significantly expanded to encompass traditional databases and newer sources such as the social media, wearables, and mobile devices. Advances in information technology, along with the growth in computational power and the evolution of analytical methods relying on bioinformatic tools and/or artificial intelligence techniques, have enhanced the potential for utilizing this data to generate real-world evidence and improve clinical practice. Indeed, these innovative analytical approaches enable the screening and analysis of large amounts of data to rapidly generate evidence. As such numerous practical uses of artificial intelligence in medicine have been successfully investigated for image processing, disease diagnosis and prediction, as well as the management of pharmacological treatments, thus highlighting the need to educate health professionals on these emerging approaches. This narrative review provides an overview of the foremost opportunities and challenges presented by artificial intelligence in pharmacology, and specifically concerning the drug post-marketing safety evaluation.

## 1 Introduction

Over the past years, there has been a significant surge in the generation of routinely collected electronic data, commonly referred to as “real-world data” (RWD), and this trend is expected to continue in the coming years.

Today, in addition to traditional data sources (e.g., claims databases, electronic health records–EHR -, drug and disease registries, and spontaneous reporting system databases), RWD come from sources that have not been deeply explored so far, including social media platforms, as well as wearable and mobile devices. All of this resulted into the swift generation of RWD, which are heterogeneous in terms of complexity, completeness, granularity, and quality and are often unstructured, thus requiring innovative analytical approaches to be analyzed and generate evidence (Crisafulli et al., 2023). Recent advances in information technology (IT) have enabled large-scale data infrastructures setting up, allowing large volume of electronic data to be securely aggregated, stored, shared, and analyzed. The increasing computational power in terms of performance, speed, and storage, as well as the development of innovative analytical approaches, based on bioinformatics tools and artificial intelligence (AI) or machine learning (ML) techniques, are expanding the possibilities for exploring unstructured data (Haug and Drazen, 2023). The exponentially increased generation of healthcare data provides a promising avenue for the development and application of AI methodologies aimed at improving clinical practice and the quality of care (Liu et al., 2021; Trifirò and Crisafulli, 2022).

AI is a wide branch of computer science aimed at developing smart machines using predictions and automation to perform tasks typically requiring human intelligence (International Business Machines Corporation, 2023a). ML is a type of AI focusing on the use of data and algorithms to emulate the way that humans learn, gradually improving its own accuracy (International Business Machines Corporation, 2023b). Deep learning (DL) is a part of ML, based on the use of neural networks of more than three layers, many times related with representation learning (International Business Machines Corporation, 2023c). The use of DL models to create texts, images, and other content by leveraging information from training datasets is referred to as generative AI. The applications of this type of AI in the healthcare sector are developing fast, and in the near future, such models (e.g., large language models–LLMs) may play a role in analyzing different types of data, including medical imaging and RWD, protein structure prediction, clinical documentation, diagnostic assistance, clinical decision support, and drug design (Shaban-Nejad et al., 2023). However, to date, generative AI has unpredictable fluctuations in terms of quality and accuracy, as well as some ethical issues that could limit its full application in the healthcare sector, thus highlighting the need for human supervision (Zhang and Kamel Boulos, 2023). In this regard, to improve generative AI, the concept of “constitutional AI” emerges as a crucial concept focused on guaranteeing that AI activities conform to the legal and ethical principles outlined in National Constitutions or other legal documents.

The evolution of Big Data and the increasing awareness of their potential to support public health and regulatory decision-making processes are leading to a growing interest from regulatory agencies in generating real-world evidence using AI approaches to analyze Big Data. In this regard, the Heads of Medicines Agencies (HMA) and the European Medicines Agency (EMA) developed a joint task force on Big Data, which formulated several recommendations, including the establishment of a platform to access and analyze healthcare data across Europe and the building of computing capacity to analyze Big Data using advanced analytics and AI (Heads of Medicines Agencies and European Medicines Agency, 2018). Based on these recommendations, at the beginning of 2022, the EMA established a Coordination Center for the Data Analysis and Real-World Interrogation Network (DARWIN EU^®^), aimed at providing evidence on the use, safety, and effectiveness of medicines using European real-world healthcare databases (European Medicines Agency, 2022).

The increasing use of advanced analytics and AI in medicine highlights the need to train health professionals by developing specific training *curricula* covering such emerging issues (Crisafulli S et al., 2022). Nowadays, the use of AI and ML techniques finds several applications in medicine, encompassing image processing, disease diagnosis, and prediction, as well as the management of chronic diseases and of pharmacological therapies. Current evidence suggests that AI-based conversational agents may be helpful for several chronic diseases (e.g., heart failure, asthma, Alzheimer, Parkinson, depression, and diabetes) by developing specialized chatbots (Singareddy et al., 2023). These tools are also increasingly being integrated with remote outpatient monitoring, especially for home therapies and follow-ups, thus facilitating the reporting of adverse events. Furthermore, the integration of chatbot technology into clinical practice may lead to cost reduction, improved patient outcomes, and higher treatment compliance rate (Xu et al., 2021; Chen et., 2023).

The aim of this narrative review is to provide an overview of the main opportunities and challenges of AI applications to all the phases of a medicine lifecycle, with a specific focus on drug post-marketing safety evaluation.

## 2 Artificial intelligence for drug discovery and repurposing, clinical trials, and digital therapeutics development

**FIGURE 1:**
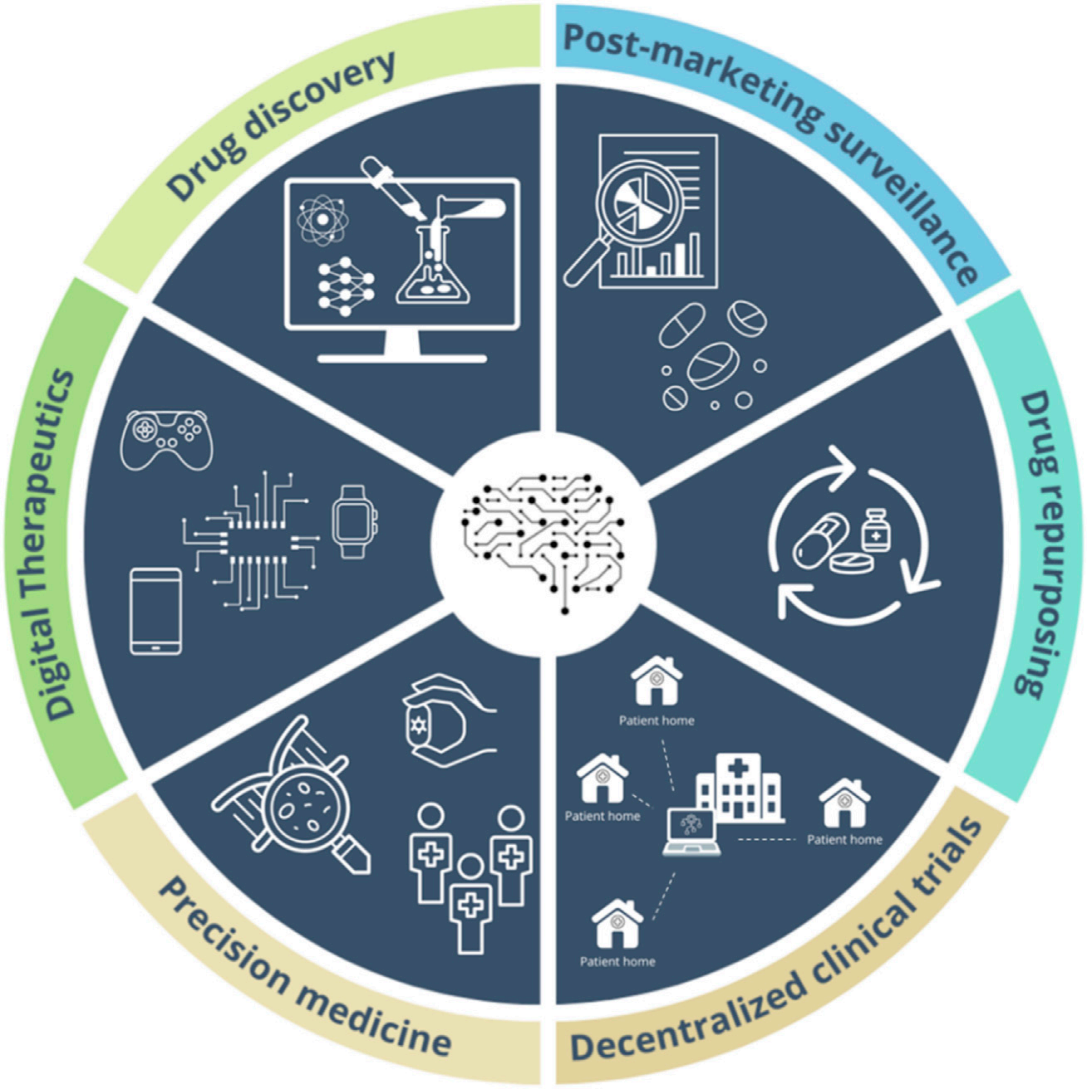
Main applications of Artificial Intelligence technologies in pharmacology.

The main applications of AI techniques in pharmacological research range from drug discovery and repurposing, clinical trials, precision medicine, and the post-marketing surveillance of drugs (Figure 1).

AI can be applied to different stages of the drug discovery process, where it can accelerate high-throughput screening (HTS), identify new biological targets, and predict the bioactivity of new drugs (Parvatikar et al., 2023). Such applications gain particular importance, especially concerning healthcare emergencies such as the antibiotic resistance (Wencewicz, 2019; World Health Organization, 2020). The application of ML methods can support computational tools in accelerating the discovery of new antibiotics by analysing large chemical and biographical datasets to design new molecules with antimicrobial activity, optimize already known drugs, as well as identify new antimicrobial resistance markers (Cesaro A et al., 2023). The application of ML techniques to rapidly screen multiple chemical libraries allowed the discovery of halicin (Stokes et al., 2020) and abaucin (Liu et al., 2023), two antibiotics which are able to inhibit the growth of *Escherichia coli* and *Acinetobacter baumannii*, respectively, in murine models. These pathogens are included in the World Health Organization’s list of antibiotic-resistant “priority pathogens”.

Furthermore, there is a growing trend toward digitizing the management and conduction of clinical trials, which are rapidly transitioning toward decentralization (Thomas and Kidziński, 2022). Decentralized clinical trials are studies reducing or eliminating the need for patients to physically reach the sites in which the trial is conducted, thus allowing patients access to trials irrespective of their geographic location (Subbiah, 2023). This includes the development of digital platforms (e.g., apps and wearable devices), the integration of clinical trial management systems and EHRs, as well as progress in digital health technologies (e.g., electronic diaries collecting electronic patient-reported outcomes, smartphones), which allow some health assessments to be remotely performed in participants’ homes (i.e., digital endpoints) (Sehrawat et al., 2023; Subbiah, 2023). The use of such technologies for the conduction of clinical trials has the advantages of providing a higher amount of high-quality data, avoiding bias related to manual data collection, reducing the dropout rate, and enhancing the enrollment of patients (Thomas and Kidziński, 2022).

AI can support the conduct of RCTs by generating synthetic data, which are data produced by algorithms trained to learn the features of real-world datasets (Van Le et al., 2023). The so-called “digital patients” are virtual copies of real patients which are created based on clinical and genomic data collected in real-world data sources. The generation of synthetic cohorts to be used as control arms holds promise to accelerate clinical research in those fields where the conduction of such studies is limited by ethical and/or feasibility issues, such as rare diseases and pediatric diseases in general (D’Amico et al., 2023).

Due to their ability to apply learning strategies built upon classification or pattern recognition to both biological and healthcare data, AI algorithms are increasingly used to generate patient-level prediction models for diagnosis, personalized therapeutic intervention, and prognosis (Reps et al., 2018; Uddin et al., 2019). This is particularly useful in oncology, where AI technologies can be used to examine omics data (e.g., proteomics, genomics, metabolomics, and epigenomics), aiming to identify diagnostic and prognostic biomarkers, detect genetic and molecular alterations, predict therapeutic outcomes, and develop targeted medicines (Picard et al., 2021).

Similarly, the considerable amount of RWD and the development of advanced AI techniques are boosting drug repurposing research, especially in the field of rare diseases, which can benefit from these analytical approaches in discovering new relationships among various types of biological entities and drugs already approved for other therapeutic indications (Fetro and Scherman, 2020; Liu et al., 2021; Yang et al., 2022). One of the first experiences of the application of AI techniques for the identification of potential treatments for rare diseases is IBM Watson^®^, a cognitive computing technology that includes medical literature, patents, genomic, chemical, and pharmacological data to make new connections in millions of pages of text. This tool has been applied to some pilot studies, facilitating a more rapid identification of new drug candidates and new drug targets by harnessing the potential of Big Data (Chen et al., 2016). Similarly, the Hugh Kaul Institute for Precision Medicine has created mediKanren, an AI-based platform that, using knowledge graphs, allows to efficiently link all relevant literature and databases to identify drugs, genetic targets, and, ultimately, potentially relevant therapeutic options for rare diseases (Foksinska et al., 2022).

AI may play a pivotal role in the development and utilization of digital therapeutics (DTx), providing a personalized and data-driven approach to patient care. A DTx is a health software intended to treat or alleviate a disease, disorder, condition, or injury by generating and delivering a medical intervention that has a demonstrable positive therapeutic impact on a patient’s health (Crisafulli et al., 2022).

AI may contribute significantly to their research, development, and clinical use in different ways:

• Personalization of treatments: AI can analyze individual data, such as medical history and lifestyle habits, to personalize treatments and maximize their effectiveness.
• Real-time monitoring: AI-enabled devices can collect real-time data from patients, enabling constant monitoring and immediate adjustment of treatments and, in some cases, creating a closed-loop therapeutic system.
• Evidence-based therapies: AI can analyze huge amounts of clinical data to identify the most effective treatments, ensuring that DTx are based on sound scientific evidence.
• Improved adherence: AI-based applications can motivate patients to follow their treatments more consistently through personalized monitoring, reminders, and feedback.

In conclusion, AI is about to play a critical role in the present and future of Digital Therapeutics, improving patient outcomes, reducing healthcare costs, and making personalized healthcare accessible to a broader population. This synergy between technology and healthcare is transforming the way we approach wellness and treatment, creating a more patient-centric and efficient healthcare ecosystem (Recchia and Gussoni, 2023). However, to date the European regulatory framework pertaining to DTx is still in its early stages, highlighting the need for *ad hoc* regulations aimed to assess these tools and guarantee their safety as well as the integrity of the data collected. Considering that the use of DTx will rapidly enter into clinical practice, post-marketing safety surveillance needs to be meticulously implemented to promptly identify potential safety issues, also by promoting the collection and analysis of real-world data (Crisafulli et al., 2022).

## 3 Main challenges of artificial intelligence for the management of pharmacological therapies

### 3.1 Prerequisites

The integration of AI in healthcare presents several challenges in developing and validating solutions to ensure reliability and generalizability across different populations (Table 1) (Futoma et al., 2020). One of the key challenges in implementing AI-based approaches is the availability of suitable data (Vamathevan et al., 2019), mainly due to issues related to data quality as well as data privacy and sharing. Healthcare data are frequently limited, inconsistent, or of poor quality (Blanco-González et al., 2023). In pivotal clinical trials, AI may need to navigate through a complex network of heterogeneous (e.g., multimodal data and multisite variability) and fragmented data (i.e., data stored in separate databases or platforms, discrepancies in data recording methods, units, and formats) (Blanco-González et al., 2023). The inherent complexity and heterogeneity of healthcare data can pose significant challenges for the development of AI models. Consequently, an AI system that performs well with a particular group of patients may not exhibit the same level of performance when applied to a different healthcare context. To address these challenges, it is essential to promote data harmonization and the development of AI models with large, representative, multicenter datasets. Indeed, most AI publications available are based on data from single centers without external validation. Especially for pharmacovigilance and pharmacoepidemiological research, the establishment of distributed data networks and the development of federated learning techniques, where data does not need to be shared but the algorithms are applied on local data, may help overcome such issues. However, such infrastructures also present some challenges for the application of AI techniques, mainly arising from practical data-related aspects, including the types of data sources used, the degree of standardization across different sites, and the granularity of data sharing among these sites (Gini et al., 2020). Examples of distributed data networks for pharmacovigilance and pharmacoepidemiological research include the European Health Data and Evidence Network (EHDEN) (European Health Data Evidence Network, 2024) and the Observational Health Data Sciences and Informatics (OHDSI) Collaborative (Hripcsak et al., 2015) in Europe, as well as the Sentinel System in the United States (Ball et al., 2016) and the Canadian Network for Observational Drug Effect Studies (CNODES) in Canada (Suissa et al., 2022).

**TABLE 1:** Main challenges and opportunities in implementing artificial intelligence in pharmacological therapies.

Opportunities	
Drug discovery and development	AI can significantly accelerate the process of drug discovery, identifying potential candidates more rapidly and efficiently than traditional methods
Personalized medicine	AI can analyze complex datasets to identify patterns, enabling more personalized and effective treatments tailored to individual patient profiles
Predictive analytics	AI can predict diagnoses, clinical outcomes, or behaviors (e.g., adverse drug reactions, drug interactions, adherence to pharmacological treatments), improving patient safety and therapy effectiveness
Enhanced decision support	AI can assist healthcare professionals in making more informed decisions by providing comprehensive, data-driven insights
Cost reduction	In the long term, AI can reduce costs by optimizing treatment regimens, minimizing adverse effects, and reducing the need for expensive diagnostic tests
Continuous learning and improvement	AI systems can continually learn from new data, leading to ongoing improvements in pharmacological treatment strategies and patient outcomes
Challenges	
Data quality and availability	AI models require large amounts of high-quality, diverse, and representative data. In pharmacology, access to such datasets can be limited due to patient privacy and data sharing concerns
Model transparency and interpretability	AI, and especially deep learning, often rely on black box models. This lack of transparency can be a significant issue in clinical settings where understanding the basis for a model’s decision is crucial
Regulatory and ethical challenges	Navigating the complex regulatory landscape for AI applications in healthcare is challenging. Ethical considerations, such as bias in data leading to unequal treatment recommendations, also need to be addressed
Integration with already existing systems	To be effective, AI algorithms must be integrated with existing pharmacological systems. This can be a major challenge due to the complexity of existing systems and the need to ensure compatibility with different data formats and standards
Skills and training	Training researchers and healthcare professionals to effectively use and interpret AI-based tools is essential for their successful adoption
Cost and resource constraints	Developing and implementing AI solutions can be resource-intensive. This includes costs related to computing infrastructure, data storage, and ongoing maintenance

Abbreviations: AI = Artificial intelligence.

Furthermore, research in the field of AI is usually the result of studies on retrospective data and is rarely prospective. Given these limitations, it is important that the performance of such systems is validated not only statistically but also from a clinical perspective. For instance, the use of AI to calculate personalized drug dosages based on variables such as age, sex, weight, and health conditions must also account for genetic variability and the biological complexity that a patient population might exhibit in a specific clinical context (Futoma et al., 2020). Therefore, it is imperative that the performance of such systems is validated not only statistically but also from a clinical perspective. A thorough evaluation of the real-world clinical effectiveness and applicability of AI systems requires the implementation of an external validation process, utilizing sufficiently large datasets sourced from institutions different from those contributing to the model’s training data (Debray et al., 2015). Establishing standards and protocols for clinical validation assures that these technologies meet the required standards for patient care without posing undue risks.

### 3.2 System architecture and design

Since the reasoning behind AI algorithms’ predictions is not straightforward, the interpretability and transparency of these systems hold crucial significance, especially for pharmacological research, where discerning the factors influencing the safety and efficacy of treatments is essential.

To make informed decisions ensuring patient safety and treatment effectiveness, physicians need to understand the clinical basis of AI recommendations for drug dosages, treatment choice, or outcome prediction. As such, AI systems in pharmacology should clearly indicate the data sources used and explain how these sources influence their conclusions. Transparency is integral to building trust among clinicians and integrating AI tools into clinical settings. The system architecture should thus incorporate mechanisms for interpretability and transparency at its core, such as rationale visualization interfaces illustrating the AI decision-making processes for clinicians in an accessible format. Additionally, ensuring the traceability of data sources is critical, allowing every AI-generated recommendation to be linked back to its original data and confirming the validity and relevance of the information used.

### 3.3 Implementation

The implementation of AI technologies in healthcare raises substantial ethical and legal concerns. Questions surrounding data privacy, consent, and security are paramount, especially given the sensitivity of health-related data (Dalton-Brown, 2020). At both the global (e.g., pharmacovigilance) and individual patient levels, the handling of sensitive data must adhere to stringent data privacy regulations. AI systems must comply with relevant governance frameworks and legal provisions safeguarding data privacy and integrity, such as the General Data Protection Regulation (GDPR) in Europe, the Health Insurance Portability and Accountability Act (HIPAA) in the United States of America, and the Personal Information Protection Law (PIPL) in China, among others.

In this regard, the European Union is about to enact the AI Act, introducing harmonized regulations for AI and establishing a risk-based classification system for its applications. Under this act, a significant number of healthcare-related AI applications will be categorized as “high-risk” due to their considerable implications for individuals’ wellbeing and fundamental rights. This high-risk designation implies stringent obligations, including but not limited to, undergoing conformity assessments, maintaining comprehensive documentation, and fulfilling registration requirements. As the AI landscape navigates these comprehensive regulations, it will be crucial for developers and stakeholders to strategize effectively to mitigate challenges such as increased costs, prolonged development cycles, and the heightened complexity of introducing AI products to the market. These factors could potentially slow the pace of AI innovation, emphasizing the need for a balanced approach that safeguards public interests while fostering technological advancement (European Union, 2024).

Furthermore, the decision-making transparency of AI models, especially in clinical settings, is crucial to guarantee the ethical use of data and to build trust among healthcare professionals and patients (Couture et al., 2023). Legal frameworks contemplating responsibility and accountability in the event of incorrect functioning of AI systems are also imperative to protect interests and secure redressal mechanisms. Obtaining informed consent in the context of AI is challenging but necessary, guaranteeing that patients and participants are adequately informed about how AI will be utilized in their care or in research contexts (Dalton-Brown, 2020). At the same time, the consent mechanism creates a selection among patients who provide data, potentially leading to biases in the final algorithm (Woolf et al., 2000). Regarding AI-based clinical trials (including decentralized clinical trials, where technology facilitates remote monitoring and data collection from participants in their usual environments), it is indispensable to consider ethical aspects related to ensuring equitable participant recruitment, maintaining data integrity, and granting participant autonomy and confidentiality (Thomas and Kidziński, 2022). Generally, there is still no consensus on the governance mechanism that should address these ethical issues, whether it be private actors and corporations or public institutions to supervise the ownership, sharing, and use of patient data in the context of pharmacological therapy and, more broadly, for the implementation of AI systems (Dalton-Brown, 2020). Recently, the FDA published two discussion papers focused on the development of drug and biological products and on the regulatory framework for modifications to AI-based software as a Medical Device (SaMD) (Food and Drug Administration, 2023a; Food and Drug Administration, 2023b). The regulatory relevance of AI is already tangible: in 2021, over 100 drug and biological product applications submitted to the FDA included AI components (Crisafulli S et al., 2022; Liu et al., 2023). The aim of the papers is to stimulate discussions among different stakeholders, including pharmaceutical companies, ethicists, academia, patients, regulatory agencies, and other authorities worldwide. The dialogue focuses on utilizing AI technologies in drug and biological product development, including the development of medical devices intended to be used with drugs, to help inform the regulatory landscape in this area. The establishment of a regulatory landscape is key to allowing further development of these powerful innovations and, ultimately, enabling their actual implementation in healthcare.

Barriers of an economic nature, including challenges related to the development and reimbursement for DTx and AI-driven interventions, may also hinder the adoption of AI in pharmacological therapy management. Developing, validating, and implementing AI solutions require substantial investments in technology, training, and possibly re-engineering of existing systems and processes. This is why clarity in the regulatory landscape and the expected requirements for the final product developed are key steps to allow for innovation and related investments. In addition, concerns about environmental sustainability have recently been raised. The energy and the massive computation required to produce and process vast amounts of data significantly impacts the environment, due to the non-renewable energy used to fuel modern data processing hardware. Furthermore, the unsustainable extraction of minerals for technology components and the disposal of electronic waste are aspects to be considered to avoid negative environmental impacts (Strubell et al., 2020; Samuel et al., 2022).

Ensuring equal access to AI-driven healthcare interventions is also crucial to avoid exacerbating existing healthcare disparities, and to preclude the inadvertent sidelining of underrepresented groups (Abràmoff et al., 2022). Sustained investments in AI technologies, tailoring AI systems to cater to diverse populations, cultures, languages, and healthcare contexts, and implementing AI systems in low- and middle-income countries would contribute to reducing global health disparities (Singh et al., 2020). Addressing these challenges in an integrated, ethical, and accessible manner is pivotal to fully realizing the potential of AI in enhancing pharmacological therapies and wider healthcare outcomes.

### 3.4 Integration with existing systems

Another issue lies in creating and developing AI systems capable of continuous learning and adaptation to evolving clinical landscapes and patient needs, by employing architectures that facilitate the scalability and adaptability of these systems. The approach should involve a constant and systematic update with the most recent information on medications, clinical studies, and guidelines (Food and Drug Administration, 2023a). The updated pharmacological data should be smoothly integrated with other existing healthcare IT infrastructures (e.g., EHRs and clinical decision support systems) in a secure and structured workflow. Indeed, AI systems should seamlessly interact with such infrastructures and other healthcare management tools for optimal functionality and user acceptance, representing a challenge for the existing technological infrastructures that are not designed for continuous updates. (Singh et al., 2020). Integration challenges also include verifying that healthcare professionals can adeptly navigate and utilize AI tools, highlighting the need for comprehensive training and change management strategies to facilitate technological transitions (Holland Brown and Bewick, 2023). To achieve these objectives, digital literacy among healthcare professionals and patients should be enhanced to support the effective use of user-friendly AI technologies, ensuring they are accessible to all users, regardless of their technical proficiency (Holland Brown and Bewick, 2023). In a future-oriented perspective, AI will likely change the way healthcare assistance is organized, and easy-to-navigate technologies are expected to improve healthcare professionals’ capabilities rather than replace them. Indeed, the correct interpretation of the data and of the results yielded by AI tools still requires human supervision to avoid errors and ensure responsible, ethical, and effective AI development and deployment.

## 4 Artificial intelligence in pharmacovigilance

The application of AI in pharmacovigilance represents a turning point in drug safety surveillance and management, with a potential impact in speeding up the detection and analysis of safety signals in a timely and effective manner. The use of advanced methodologies, including ML techniques, as well as the access to large amounts of electronic health data are increasingly improving and have the potential to accelerate the assessment of the risk-benefit profile of drugs in the real-world setting. In recent years, public health emergencies, such as the COVID-19 pandemic, as well as the marketing of innovative therapies (e.g., advanced therapy medicinal products, DTx, and vaccines based on advanced technologies) with accelerated approval highlight the importance of proactive surveillance, and the need to rapidly generate safety post-marketing data, identifying and preventing serious risks (Trifirò and Crisafulli, 2022).

The main AI methodologies supporting pharmacovigilance activities include natural language processing (NLP) and text mining techniques, which can extract useful information from large streams of data from structured and unstructured sources, identifying patterns and correlations that would otherwise be difficult to determine through conventional methods (Marella et al., 2017; Evans et al., 2020). Yang and others demonstrated that the application of a DL model to free-text narrative of hospital safety event reports improved the accuracy of the identification of allergic reactions. Indeed, as compared with traditional approaches, the DL model identified 24% more cases of confirmed allergic reactions and reduced the need for manual review by 64% (Yang J et al., 2020).

Although such technologies are not routinely used for pharmacovigilance activities, they have shown a promising role in collecting information on adverse drug reactions (ADRs) and drug-drug interactions from various textual sources (Bouzillé et al., 2019; Ben Abacha et al., 2015), supporting researchers and clinicians in monitoring drug safety (Wong et al., 2018) and assessing the causality of drug events in individual case safety reports (ICSRs) (Comfort S et al., 2018).

The application of AI in pharmacovigilance is also paving the way for a more personalized approach to patient safety evaluation by enabling the identification of clusters of adverse events that represent syndromes attributable to drugs and the prediction and prevention of ADRs through the creation of RWD-based predictive models (Bate and Hobbiger, 2021), and the estimation of potential risks associated with the use of drugs, ultimately helping health professionals make informed decisions (Dewulf P et al., 2021). In this regard, Wang et al. developed a knowledge graph by applying ML techniques to the biomedical literature databases, to discover potential antineoplastic-related ADRs based on biomarkers. This tool is able to identify relationships among four types of nodes (i.e., tumor, biomarker, drug, and ADR). Using the example of osimertinib, the authors demonstrated that this model achieved high accuracy in discovering both known and potentially new ADRs, thus providing oncologists with the opportunity to quickly predict the susceptibility of patients to antitumoral-related ADRs (Wang M et al., 2021). Furthermore, ML and DL techniques have been also extensively used for the automated detection and prediction of ADRs using social media, which, however, pose several challenges for the application of AI techniques, including the sparseness of adverse drug events, and the unstructured texts of social media posts (Huang et al., 2022). Advances in AI increasingly allow us to effectively overcome these issues. For example, the use of different techniques to address data imbalances (e.g., resampling and ensemble learning), and data heterogeneity (e.g., feature selection), as well as the application of a DL-based approach adopting the Bidirectional Encoder Representations from Transformers (BERT) model to Twitter were found to be effective in enhancing ADR prediction using social media (Huang et al., 2022).

Given that information on potential ADRs is mostly hidden in EHRs, the scientific literature reports many experiences in which ML models were able to capture such data from these sources, allowing more accurate classification of patients, minimization of risks, better care of patients as well as easier management of complex therapies.

Therefore, ML can be used to identify populations at high risk of developing ADRs, determine the severity of ADRs (Routray et al., 2020), as well as accurately identify patients most susceptible to the toxic effects of specific medicines (Correia Pinheiro L et al., 2020). A clear application of ML to achieve these objectives is AwareDX, a pharmacovigilance algorithm using pharmacogenomic data to predict the sex-specific risk of experiencing ADRs (Chandak and Tatonetti, 2020). This algorithm assessed whether a drug-ADR pair has a sex-significant risk in three steps: 1) computation of propensity scores for each patient to minimize bias and confounding; 2) construction of sex-balanced cohorts for each subpopulation of drug users, and 3) application of disproportionality analysis to the balanced cohorts to quantify the sex-related risk of drug-related ADR. Applied to the FDA Adverse Event Reporting System (FAERS) database, AwareDX was able to identify sex-specific ADRs, many of which were previously unknown. In this way, this model may be helpful for minimizing ADRs associated with specific drugs by tailoring drug prescribing and dosing to patients’ sex (Chandak and Tatonetti, 2020).

However, more experiences and applications of AI in pharmacovigilance and post-marketing drug safety monitoring are needed before these methods can be validated and promoted for widespread use.

When used properly and in line with current pharmacovigilance best practice requirements, AI tools can effectively support the acquisition, transformation, analysis, and interpretation of large amounts of pharmacovigilance data; therefore, these systems could follow the same regulatory pathways of other pharmacological interventions and healthcare devices.

## 5 Artificial intelligence in pharmacoepidemiology

In recent years, AI has also emerged as a powerful tool for pharmacoepidemiological research, where it can help address challenges such as data complexity, scale, and the need for real-time analysis. AI algorithms can rapidly and accurately analyze a wide range of real-world data sources (e.g., claims databases, EHR, and drug/disease-specific registries) and distributed database networks to identify patterns and relationships that would be challenging or impossible to detect using traditional epidemiological methods (Wong et al., 2022).

AI, and ML in particular, can significantly aid in conducting several activities pertaining the conduction of pharmacoepidemiological studies, including phenotyping, causal inference, the prediction of diagnosis and clinical response, and the conduction of drug utilization analyses.

In pharmacoepidemiology, a “phenotype” is a measurable biological, behavioral, or clinical marker of a condition or disease, that can be identified by applying computerized algorithms to a defined set of health data. Although such algorithms are usually developed by experts based on their knowledge of the clinical condition under study, the data driven approach of ML algorithms can be useful to explore a large number of clinical features, allowing the identification of potentially latent associations, or predictors, as well as the development of new phenotypes (Liu et al., 2021).

Similarly, ML models are increasingly used to address imbalances in risk factors between the study cohorts and for the evaluation of causal effects in observational studies performed on healthcare databases. The theoretical benefits of employing ML models lie in their automated processes that eliminate the necessity for researcher-defined covariate selection, as well as in the flexible modeling of non-linear effects and interactions (Karim et al., 2018). As compared to traditional pharmacoepidemiological approaches, AI models, such as artificial neural networks, random forest algorithms, and Hybrid-least absolute shrinkage and selection operator (LASSO), were found to provide less biased estimates of the propensity scores (Webster-Clark et al., 2021; Rassen et al., 2023), as well as to yield better performances for the identification of confounders within the framework of high-dimensional propensity scores (Schneeweiss et al., 2009; Karim et al., 2018).

Another application of AI in pharmacoepidemiology concerns the prediction of diagnoses or clinical outcomes using large-scale, real-world data sources. ML can be used to develop accurate prognostic models using historical data to forecast health outcomes, through the identification of latent and complex associations among a large number of clinical features. Such models play a key role in helping clinicians plan early interventions and make informed decisions. A considerable number of examples of ML-based prediction models relying on claims databases are reported in the scientific literature, including models for the prediction of the diagnosis of rare diseases (Ong et al., 2020; Hyde et al., 2023), models for the prediction of disease activity (Norgeot et al., 2019), as well as models to forecast the risk of different clinical events, such as penicillin allergy (Gonzalez-Estrada et al., 2024), suicidal behavior (Simon et al., 2024), hospital readmissions (Huang et al., 2021), and healthcare costs (Vimont et al., 2022).

Within this context, ML and data mining techniques can be effectively used also to predict drug utilization and guide clinical decisions for improving health outcomes and the quality of care (Huang et al., 2021).

In particular, the application of a random forest model on a claims database was used to predict variations in treatment patterns in patients affected by epilepsy, providing good predictive power. More recently, analyzing electronic medical records, Liu et al. demonstrated that, compared with the logistic regression model, the random forest algorithm yielded a better performance in predicting the switch of disease-modifying agents among patients with multiple sclerosis (Li et al., 2023).

Lastly, ML models can be used to identify predictors of treatment persistence and adherence and the prediction of medication adherence thresholds. As an example, Hackshaw et al. used a random forest algorithm on claims databases to identify younger age and higher comorbidity index as predictors of pazopanib persistence and adherence in naïve patients (Hackshaw et al., 2014). Similarly, a Bayesian network was used to compare and evaluate potential predictors of adherence and symptom remission within a cohort of schizophrenic patients treated with paliperidone palmitate as compared to those receiving other atypical oral antipsychotics (Anderson et al., 2017). In addition, the application of a Bayesian random survival forest model to the Medicaid claims database allowed the identification of adherence thresholds for optimal discrimination of hospitalization risk in patients with type 2 diabetes (Lo-Ciganic et al., 2015).

As AI technologies evolve, ongoing research and collaboration are essential to harness their full potential and address the challenges associated with their implementation for the conduction of real-world studies.

## 6 Conclusion

In the near future, AI technologies are expected to be increasingly used in supporting healthcare activities as well as all the phases of the medicinal products lifecycle. The exponential growth of big data infrastructures and the availability of advanced technologies based on AI are enabling the collection, transformation, analysis, and interpretation of this large volume of data for timely and reliable evidence generation with the final goal of improving clinical practice and quality of care and ensuring patients’ safety. The advancement of AI-driven technologies signifies a paradigm shift, especially evident in the enhanced precision of diagnostic procedures, the post-marketing safety evaluation, the personalization of therapeutic approaches, and the deepening of our understanding of complex disease pathways. In such rapidly changing scenarios, humans have a central and indispensable role in the proper and ethical use of these technologies and in the correct interpretation of the evidence generated, highlighting the need to train health professionals on such emerging issues. In this regard, as AI technologies continue to rapidly evolve, it is crucial that healthcare stakeholders and end-users keep a balance between AI efficiency and control, thus ensuring a responsible AI implementation in pharmacological research. Especially in the field of pharmacovigilance and pharmacoepidemiological research, the implementation of privacy-preserving techniques (e.g., differential privacy, federated learning, and homomorphic encryption) can help protect sensitive data and preserve individuals’ privacy rights in AI applications. These techniques enable data to be used for training AI models without exposing sensitive information to unauthorized parties. Lastly, the forthcoming years will be critical in shaping regulatory policies and research trajectories that embrace these innovations while upholding the highest standards of ethical and clinical practice.

## References

[B1] Abràmoff M. D. Roehrenbeck C. Trujillo S. Goldstein J. Graves A. S. Repka M. X. (2022). A reimbursement framework for artificial intelligence in healthcare. NPJ Digit. Med. 5 (1), 72. 10.1038/s41746-022-00621-w 35681002 PMC9184542

[B2] Anderson J. P. Icten Z. Alas V. Benson C. Joshi K. (2017). Comparison and predictors of treatment adherence and remission among patients with schizophrenia treated with paliperidone palmitate or atypical oral antipsychotics in community behavioral health organizations. BMC Psychiatry 17, 346. 10.1186/s12888-017-1507-8 29047368 PMC5648472

[B3] Ball R. Robb M. Anderson S. A. Dal Pan G. (2016). The FDA's sentinel initiative--A comprehensive approach to medical product surveillance. Clin. Pharmacol. Ther. 99 (3), 265–268. 10.1002/cpt.320 26667601

[B4] Bate A. Hobbiger S. F. (2021). Artificial intelligence, real-world automation and the safety of medicines. Drug Saf. 44 (2), 125–132. 10.1007/s40264-020-01001-7 33026641

[B5] Ben Abacha A. Chowdhury M. F. M. Karanasiou A. Mrabet Y. Lavelli A. Zweigenbaum P. (2015). Text mining for pharmacovigilance: using machine learning for drug name recognition and drug-drug interaction extraction and classification. J. Biomed. Inf. 58, 122–132. 10.1016/j.jbi.2015.09.015 26432353

[B6] Blanco-González A. Cabezón A. Seco-González A. Conde-Torres D. Antelo-Riveiro P. Piñeiro Á. (2023). The role of AI in drug discovery: challenges, opportunities, and strategies. Pharm. Basel, Switz. 16 (6), 891. 10.3390/ph16060891 PMC10302890 37375838

[B7] Bouzillé G. Morival C. Westerlynck R. Lemordant P. Chazard E. Lecorre P. (2019). An automated detection system of drug-drug interactions from electronic patient records using big data analytics. Stud. health Technol. Inf. 264, 45–49. 10.3233/SHTI190180 31437882

[B8] Cesaro A. Bagheri M. Torres M. Wan F. de la Fuente-Nunez C. (2023). Deep learning tools to accelerate antibiotic discovery. Expert Opin. drug Discov. 18 (11), 1245–1257. 10.1080/17460441.2023.2250721 37794737 PMC10790350

[B9] Chandak P. Tatonetti N. P. (2020). Using machine learning to identify adverse drug effects posing increased risk to women. Patterns (New York, N.Y.) 1 (7), 100108. 10.1016/j.patter.2020.100108 33179017 PMC7654817

[B10] Chen N. J. Huang C. M. Fan C. C. Lu L. T. Lin F. H. Liao J. Y. (2023). User evaluation of a chat-based instant messaging support health education program for patients with chronic kidney disease: preliminary findings of a formative study. JMIR Form. Res. 7, e45484. 10.2196/45484 37725429 PMC10548329

[B11] Chen Y. Elenee Argentinis J. D. Weber G. (2016). IBM Watson: how cognitive computing can Be applied to big data challenges in life sciences research. Clin. Ther. 38 (4), 688–701. 10.1016/j.clinthera.2015.12.001 27130797

[B12] Comfort S. Dorrell D. Meireis S. Fine J. (2018). MOdified NARanjo causality scale for ICSRs (MONARCSi): a decision support tool for safety scientists. Drug Saf. 41 (11), 1073–1085. 10.1007/s40264-018-0690-y 29876835 PMC6182464

[B13] Correia Pinheiro L. Durand J. Dogné J. M. (2020). An application of machine learning in pharmacovigilance: estimating likely patient genotype from phenotypical manifestations of fluoropyrimidine toxicity. Clin. Pharmacol. Ther. 107 (4), 944–947. 10.1002/cpt.1789 31955411 PMC7158217

[B14] Couture V. Roy M. C. Dez E. Laperle S. Bélisle-Pipon J. C. (2023). Ethical implications of artificial intelligence in population health and the public's role in its governance: perspectives from a citizen and expert panel. J. Med. Internet Res. 25, e44357. 10.2196/44357 37104026 PMC10176139

[B15] Crisafulli S. Khan Z. Karatas Y. Tuccori M. Trifirò G. (2023). An overview of methodological flaws of real-world studies investigating drug safety in the post-marketing setting. Expert Opin. drug Saf. 22 (5), 373–380. 10.1080/14740338.2023.2219892 37243676

[B16] Crisafulli S. Santoro E. Recchia G. Trifirò G. (2022). Digital therapeutics in perspective: from regulatory challenges to post-marketing surveillance. Front. Drug Saf. Regul. 2, 900946. 10.3389/fdsfr.2022.900946

[B17] Dalton-Brown S. (2020). The ethics of medical AI and the physician-patient relationship. Camb. Q. Healthc. ethics CQ Int. J. Healthc. ethics committees 29 (1), 115–121. 10.1017/S0963180119000847 31858938

[B18] D'Amico S. Dall'Olio D. Sala C. Dall'Olio L. Sauta E. Zampini M. (2023). Synthetic data generation by artificial intelligence to accelerate research and precision medicine in hematology. JCO Clin. cancer Inf. 7, e2300021. 10.1200/CCI.23.00021 PMC10569771 37390377

[B19] Debray T. P. Vergouwe Y. Koffijberg H. Nieboer D. Steyerberg E. W. Moons K. G. (2015). A new framework to enhance the interpretation of external validation studies of clinical prediction models. J. Clin. Epidemiol. 68 (3), 279–289. 10.1016/j.jclinepi.2014.06.018 25179855

[B20] Dewulf P. Stock M. De Baets B. (2021). Cold-start problems in data-driven prediction of drug-drug interaction effects. Pharm. Basel, Switz. 14 (5), 429. 10.3390/ph14050429 PMC8147651 34063324

[B21] European Health Data Evidence Network (2024). Available at: https://www.ehden.eu/(Accessed February 26, 2024).

[B22] European Medicines Agency (2022). Initiation of Darwin EU® Coordination Centre advances integration of real-world evidence into assessment of medicines. Available at: https://www.ema.europa.eu/en/news/initiation-darwin-eur-coordination-centre-advances-integration-real-world-evidence-assessment.

[B23] Evans H. P. Anastasiou A. Edwards A. Hibbert P. Makeham M. Luz S. (2020). Automated classification of primary care patient safety incident report content and severity using supervised machine learning (ML) approaches. Health Inf. J. 26 (4), 3123–3139. 10.1177/1460458219833102 30843455

[B75] European Union (2024). Regulation on artificial intelligence. Available at: https://data.consilium.europa.eu/doc/document/ST-5662-2024-INIT/en/pdf.

[B24] Fetro C. Scherman D. (2020). Drug repurposing in rare diseases: myths and reality. Therapie 75 (2), 157–160. 10.1016/j.therap.2020.02.006 32241561

[B25] Foksinska A. Crowder C. M. Crouse A. B. Henrikson J. Byrd W. E. Rosenblatt G. (2022). The precision medicine process for treating rare disease using the artificial intelligence tool mediKanren. Front. Artif. Intell. 5, 910216. 10.3389/frai.2022.910216 36248623 PMC9562701

[B26] Food and Drug Administration (2023a). Using artificial intelligence and machine learning in the development of drug and biological products. Available at: https://www.fda.gov/media/167973/download.

[B27] Food and Drug Administration (2023b). Artificial intelligence in drug manufacturing. Available at: https://www.fda.gov/media/165743/download.

[B28] Futoma J. Simons M. Panch T. Doshi-Velez F. Celi L. A. (2020). The myth of generalisability in clinical research and machine learning in health care. Digit. health 2 (9), e489–e492. 10.1016/S2589-7500(20)30186-2 PMC7444947 32864600

[B29] Gini R. Sturkenboom M. C. J. Sultana J. Cave A. Landi A. Pacurariu A. (2020). Different strategies to execute multi-database studies for medicines surveillance in real-world setting: a reflection on the European model. Clin. Pharmacol. Ther. 108 (2), 228–235. 10.1002/cpt.1833 32243569 PMC7484985

[B30] Gonzalez-Estrada A. Park M. A. Accarino J. J. O. Banerji A. Carrillo-Martin I. D'Netto M. E. (2024). Predicting penicillin allergy: a United States multicenter retrospective study. J. allergy Clin. Immunol., S2213-2198(24)00062-X. 10.1016/j.jaip.2024.01.010 38242531

[B32] Hackshaw M. D. Nagar S. P. Parks D. C. Miller L.-A. N. (2014). Persistence and compliance with pazopanib in patients with advanced renal cell carcinoma within a U.S. administrative claims database. J. Manage. Care Spec. Pharm. 20, 603–610. 10.18553/jmcp.2014.20.6.603 PMC10438225 24856598

[B33] Haug C. J. Drazen J. M. (2023). Artificial intelligence and machine learning in clinical medicine, 2023. N. Engl. J. Med. 388 (13), 1201–1208. 10.1056/NEJMra2302038 36988595

[B34] Heads of Medicines Agencies, European Medicines Agency (2018). HMA- EMA joint big data taskforce phase II report: “evolving data-driven regulation.”. Available at: https://www.ema.europa.eu/en/documents/other/hma-ema-joint-big-datataskforce-phase-ii-report-evolving-data-drivenregulation_en.pdf.

[B35] Holland Brown T. M. Bewick M. (2023). Digital health education: the need for a digitally ready workforce. Educ. Pract. Ed. 108 (3), 214–217. 10.1136/archdischild-2021-322022 PMC10313993 35697475

[B36] Hripcsak G. Duke J. D. Shah N. H. Reich C. G. Huser V. Schuemie M. J. (2015). Observational health data sciences and Informatics (OHDSI): opportunities for observational researchers. Stud. health Technol. Inf. 216, 574–578. PMC4815923 26262116

[B37] Huang J. Y. Lee W. P. Lee K. D. (2022). Predicting adverse drug reactions from social media posts: data balance, feature selection and deep learning. Healthc. Basel, Switz. 10 (4), 618. 10.3390/healthcare10040618 PMC9024774 35455795

[B38] Huang Y. Talwar A. Chatterjee S. Aparasu R. R. (2021). Application of machine learning in predicting hospital readmissions: a scoping review of the literature. BMC Med. Res. Methodol. 21 (1), 96. 10.1186/s12874-021-01284-z 33952192 PMC8101040

[B39] Hyde B. Paoli C. J. Panjabi S. Bettencourt K. C. Bell Lynum K. S. Selej M. (2023). A claims-based, machine-learning algorithm to identify patients with pulmonary arterial hypertension. Pulm. Circ. 13 (2), e12237. 10.1002/pul2.12237 37287599 PMC10243208

[B40] International Business Machines Corporation (2023c). What is deep learning? Available at: https://www.ibm.com/topics/deep-learning.

[B53] International Business Machines Corporation (2023a). What is artificial intelligence? Available at: https://www.ibm.com/topics/artificial-intelligence.

[B41] International Business Machines Corporation (2023b). What is machine learning? Available at: https://www.ibm.com/topics/machine-learning#:∼:text=Machine%20learning%20is%20a%20branch,rich%20history%20with%20machine%20learning.

[B42] Karim M. E. Pang M. Platt R. W. (2018). Can we train machine learning methods to outperform the high-dimensional propensity score algorithm? Epidemiology 29, 191–198. 10.1097/EDE.0000000000000787 29166301

[B44] Li J. Huang Y. Hutton G. J. Aparasu R. R. (2023). Assessing treatment switch among patients with multiple sclerosis: a machine learning approach. Explor. Res. Clin. Soc. Pharm. 11, 100307. 10.1016/j.rcsop.2023.100307 37554927 PMC10405092

[B46] Liu G. Catacutan D. B. Rathod K. Swanson K. Jin W. Mohammed J. C. (2023). Deep learning-guided discovery of an antibiotic targeting Acinetobacter baumannii. Nat. Chem. Biol. 19, 1342–1350. 10.1038/s41589-023-01349-8 37231267

[B48] Liu Q. Huang R. Hsieh J. Zhu H. Tiwari M. Liu G. (2023). Landscape analysis of the application of artificial intelligence and machine learning in regulatory submissions for drug development from 2016 to 2021. Clin. Pharmacol. Ther. 113 (4), 771–774. 10.1002/cpt.2668 35707940

[B49] Liu R. Wei L. Zhang P. (2021). A deep learning framework for drug repurposing via emulating clinical trials on real-world patient data. Nat. Mach. Intell. 3 (1), 68–75. 10.1038/s42256-020-00276-w 35603127 PMC9119409

[B50] Lo-Ciganic W.-H. Donohue J. M. Thorpe J. M. Perera S. Thorpe C. T. Marcum Z. A. (2015). Using machine learning to examine medication adherence thresholds and risk of hospitalization. Med. Care 53, 720–728. 10.1097/MLR.0000000000000394 26147866 PMC4503478

[B51] Marella W. M. Sparnon E. Finley E. (2017). Screening electronic health record-related patient safety reports using machine learning. J. patient Saf. 13 (1), 31–36. 10.1097/PTS.0000000000000104 24721977

[B52] Norgeot B. Glicksberg B. S. Trupin L. Lituiev D. Gianfrancesco M. Oskotsky B. (2019). Assessment of a deep learning model based on electronic health record data to forecast clinical outcomes in patients with rheumatoid arthritis. JAMA Netw. open 2 (3), e190606. 10.1001/jamanetworkopen.2019.0606 30874779 PMC6484652

[B54] Ong M. S. Klann J. G. Lin K. J. Maron B. A. Murphy S. N. Natter M. D. (2020). Claims-based algorithms for identifying patients with pulmonary hypertension: a comparison of decision rules and machine-learning approaches. J. Am. Heart Assoc. 9 (19), e016648. 10.1161/JAHA.120.016648 32990147 PMC7792386

[B55] Parvatikar P. P. Patil S. Khaparkhuntikar K. Patil S. Singh P. K. Sahana R. (2023). Artificial intelligence: machine learning approach for screening large database and drug discovery. Antivir. Res. 220, 105740. 10.1016/j.antiviral.2023.105740 37935248

[B56] Picard M. Scott-Boyer M. P. Bodein A. Périn O. Droit A. (2021). Integration strategies of multi-omics data for machine learning analysis. Comput. Struct. Biotechnol. J. 19, 3735–3746. 10.1016/j.csbj.2021.06.030 34285775 PMC8258788

[B57] Rassen J. A. Blin P. Kloss S. Neugebauer R. S. Platt R. W. Pottegård A. (2023). High-dimensional propensity scores for empirical covariate selection in secondary database studies: planning, implementation, and reporting. Pharmacoepidemiol Drug Saf. 32 (2), 93–106. 10.1002/pds.5566 36349471 PMC10099872

[B58] Recchia G. Gussoni G. (2023). “Digital therapeutics: scientific, technological, and regulatory challenges,” in Personalized medicine meets artificial intelligence. Editors Cesario A. D'Oria M. Auffray C. Scambia G. (Cham: Springer). 10.1007/978-3-031-32614-1_4

[B59] Reps J. M. Schuemie M. J. Suchard M. A. Ryan P. B. Rijnbeek P. R. (2018). Design and implementation of a standardized framework to generate and evaluate patient-level prediction models using observational healthcare data. J. Am. Med. Inf. Assoc. JAMIA 25 (8), 969–975. 10.1093/jamia/ocy032 PMC6077830 29718407

[B60] Routray R. Tetarenko N. Abu-Assal C. Mockute R. Assuncao B. Chen H. (2020). Application of augmented intelligence for pharmacovigilance case seriousness determination. Drug Saf. 43 (1), 57–66. 10.1007/s40264-019-00869-4 31605285 PMC6965337

[B61] Samuel G. Lucassen A. M. (2022). The environmental sustainability of data-driven health research: a scoping review. Digit. Health 8, 20552076221111297. 10.1177/20552076221111297 35847526 PMC9277423

[B62] Schneeweiss S. Rassen J. A. Glynn R. J. Avorn J. Mogun H. Brookhart M. A. (2009). High-dimensional propensity score adjustment in studies of treatment effects using health care claims data. Epidemiology 20, 512–522. 10.1097/EDE.0b013e3181a663cc 19487948 PMC3077219

[B63] Sehrawat O. Noseworthy P. A. Siontis K. C. Haddad T. C. Halamka J. D. Liu H. (2023). Data-driven and technology-enabled trial innovations toward decentralization of clinical trials: opportunities and considerations. Mayo Clin. Proc. 98 (9), 1404–1421. 10.1016/j.mayocp.2023.02.003 37661149

[B64] Shaban-Nejad A. Michalowski M. Bianco S. (2023). Creative and generative artificial intelligence for personalized medicine and healthcare: hype, reality, or hyperreality? Exp. Biol. Med. (Maywood, N.J.) 248 (24), 2497–2499. 10.1177/15353702241226801 PMC10854468 38311873

[B65] Simon G. E. Shortreed S. M. Johnson E. Yaseen Z. S. Stone M. Mosholder A. D. (2024). Predicting risk of suicidal behavior from insurance claims data vs. linked data from insurance claims and electronic health records Pharmacoepidemiol. drug Saf. 33 (1), e5734. 10.1002/pds.5734 38112287 PMC10843611

[B66] Singareddy S. Sn V. P. Jaramillo A. P. Yasir M. Iyer N. Hussein S. (2023). Artificial intelligence and its role in the management of chronic medical conditions: a systematic review. Cureus 15 (9), e46066. 10.7759/cureus.46066 37900468 PMC10607642

[B67] Singh R. P. Hom G. L. Abramoff M. D. Campbell J. P. Chiang M. F. AAO Task Force on Artificial Intelligence (2020). Current challenges and barriers to real-world artificial intelligence adoption for the healthcare system, provider, and the patient. Transl. Vis. Sci. Technol. 9 (2), 45. 10.1167/tvst.9.2.45 PMC7443115 32879755

[B68] Stokes J. M. Yang K. Swanson K. Jin W. Cubillos-Ruiz A. Donghia N. M. (2020). A deep learning approach to antibiotic discovery. Cell 181 (2), 475–483. 10.1016/j.cell.2020.04.001 32302574

[B69] Strubell E. Ganesh A. McCallum A. (2020). Energy and policy considerations for modern deep learning research. Proc. AAAI Conf. Artif. Intell. 34 (09), 13693–13696. 10.1609/aaai.v34i09.7123

[B70] Subbiah V. (2023). The next generation of evidence-based medicine. Nat. Med. 29 (1), 49–58. 10.1038/s41591-022-02160-z 36646803

[B71] Suissa S. Henry D. Caetano P. Dormuth C. R. Ernst P. Hemmelgarn B. (2012). CNODES: the Canadian network for observational drug effect studies. Indep. open-access J. 6 (4), e134–e140. PMC3654509 23687528

[B72] Thomas K. A. Kidziński Ł. (2022). Artificial intelligence can improve patients' experience in decentralized clinical trials. Nat. Med. 28 (12), 2462–2463. 10.1038/s41591-022-02034-4 36266513

[B73] Trifirò G. Crisafulli S. (2022). A new era of pharmacovigilance: future challenges and opportunities. Front. Drug Saf. Regul. 2, 2. 10.3389/fdsfr.2022.866898

[B74] Uddin M. Wang Y. Woodbury-Smith M. (2019). Artificial intelligence for precision medicine in neurodevelopmental disorders. NPJ Digit. Med. 2, 112. 10.1038/s41746-019-0191-0 31799421 PMC6872596

[B76] Vamathevan J. Clark D. Czodrowski P. Dunham I. Ferran E. Lee G. (2019). Applications of machine learning in drug discovery and development. Nat. Rev. Drug Discov. 18 (6), 463–477. 10.1038/s41573-019-0024-5 30976107 PMC6552674

[B77] Van Le H. Van Naarden Braun K. Nowakowski G. S. Sermer D. Radford J. Townsend W. (2023). Use of a real-world synthetic control arm for direct comparison of lisocabtagene maraleucel and conventional therapy in relapsed/refractory large B-cell lymphoma. Leukemia lymphoma 64 (3), 573–585. 10.1080/10428194.2022.2160200 36755418

[B78] Vimont A. Leleu H. Durand-Zaleski I. (2022). Machine learning versus regression modelling in predicting individual healthcare costs from a representative sample of the nationwide claims database in France. Eur. J. health Econ. HEPAC health Econ. Prev. care 23 (2), 211–223. 10.1007/s10198-021-01363-4 34373958

[B79] Wang M. Ma X. Si J. Tang H. Wang H. Li T. (2021). Adverse drug reaction discovery using a tumor-biomarker knowledge graph. Front. Genet. 11, 625659. 10.3389/fgene.2020.625659 33584816 PMC7873847

[B80] Webster-Clark M. Stürmer T. Wang T. Man K. Marinac-Dabic D. Rothman K. J. (2021). Using propensity scores to estimate effects of treatment initiation decisions: state of the science. Stat. Med. 40 (7), 1718–1735. 10.1002/sim.8866 33377193

[B81] Wencewicz T. A. (2019). Crossroads of antibiotic resistance and biosynthesis. J. Mol. Biol. 431 (18), 3370–3399. 10.1016/j.jmb.2019.06.033 31288031 PMC6724535

[B82] Wong A. Plasek J. M. Montecalvo S. P. Zhou L. (2018). Natural Language processing and its implications for the future of medication safety: a narrative review of recent advances and challenges. Pharmacotherapy 38 (8), 822–841. 10.1002/phar.2151 29884988

[B83] Wong J. Prieto-Alhambra D. Rijnbeek P. R. Desai R. J. Reps J. M. Toh S. (2022). Applying machine learning in distributed data networks for pharmacoepidemiologic and pharmacovigilance studies: opportunities, challenges, and considerations. Drug Saf. 45, 493–510. 10.1007/s40264-022-01158-3 35579813 PMC9112258

[B84] Woolf S. H. Rothemich S. F. Johnson R. E. Marsland D. W. (2000). Selection bias from requiring patients to give consent to examine data for health services research. Archives Fam. Med. 9 (10), 1111–1118. 10.1001/archfami.9.10.1111 11115216

[B85] World Health Organization (2020). Antibiotic resistance. Key facts. Available at: https://www.who.int/news-room/fact-sheets/detail/antibiotic-resistance.

[B86] Xu L. Sanders L. Li K. Chow J. C. L. (2021). Chatbot for health care and oncology applications using artificial intelligence and machine learning: systematic review. JMIR cancer 7 (4), e27850. 10.2196/27850 34847056 PMC8669585

[B87] Yang F. Zhang Q. Ji X. Zhang Y. Li W. Peng S. (2022). Machine learning applications in drug repurposing. Interdiscip. Sci. Comput. life Sci. 14 (1), 15–21. 10.1007/s12539-021-00487-8 PMC8783773 35066811

[B88] Yang J. Wang L. Phadke N. A. Wickner P. G. Mancini C. M. Blumenthal K. G. (2020). Development and validation of a deep learning model for detection of allergic reactions using safety event reports across hospitals. JAMA Netw. open 3 (11), e2022836. 10.1001/jamanetworkopen.2020.22836 33196805 PMC7670315

[B90] Zhang P. Kamel Boulos M. N. (2023). Generative AI in medicine and healthcare: promises, opportunities and challenges. Future Internet 15, 286. 10.3390/fi15090286

